# Partisan belief in new misinformation is resistant to accuracy incentives

**DOI:** 10.1093/pnasnexus/pgae506

**Published:** 2024-11-11

**Authors:** Jonas Stein, Marc Keuschnigg, Arnout van de Rijt

**Affiliations:** Faculty of Behavioural and Social Sciences, University of Groningen, Groningen, The Netherlands; Institute of Sociology, Leipzig University, Leipzig, Germany; Institute for Analytical Sociology, Linköping University, Norrköping, Sweden; Department of Political and Social Sciences, European University Institute, Fiesole, Italy; Department of Sociology, Utrecht University, Utrecht, The Netherlands

**Keywords:** misinformation, social media, partisanship, online experiments

## Abstract

One explanation for why people accept ideologically welcome misinformation is that they are insincere. Consistent with the insincerity hypothesis, past experiments have demonstrated that bias in the veracity assessment of publicly reported statistics and debunked news headlines often diminishes considerably when accuracy is incentivized. Many statements encountered online, however, constitute previously unseen claims that are difficult to evaluate the veracity of. We hypothesize that when confronted with unfamiliar content, unsure partisans will form sincere beliefs that are ideologically aligned. Across three experimental studies, 1,344 conservative and liberal US participants assessed the veracity of 20 politically sensitive statements that either confirmed or contradicted social science evidence only known to experts. As hypothesized, analyses show that incentives failed to correct most ideological differences in the perceived veracity of statements. Sixty six to 78% of partisan differences in accuracy assessment persisted even when monetary stakes were raised beyond levels in prior studies. Participants displayed a surprising degree of confidence in their erroneous beliefs, as bias was not reduced when participants could safely avoid rating statements they were unsure about, without monetary loss. These findings suggest limits to the ability of disciplining interventions to reduce the expression of false statements, because many of the targeted individuals sincerely believe them to be true.

**Significance Statement**

Liberals and conservatives disagree on basic facts and regularly misgauge the accuracy of politicized information. Is this because they hold fundamentally different beliefs or are they just pretending? We study how experimental participants rate the veracity of new messages they never saw before, in the presence or absence of a monetary incentive for accuracy. Results suggest that ideological differences in beliefs cannot be fully eliminated by incentives and hamper individuals’ ability to identify true from false content. Our experiments reveal that much belief in misinformation is sincere and thus immune to policy measures that increase accountability. The misinformation policy challenge is therefore not only one of inducing good behavior but also one of correcting false beliefs.

## Introduction

Even though only a small fraction of consumed news originates from hyperpartisan and fraudulent organizations (1, 2), much misinformation is less systematically generated and propagated (3–5): Ordinary partisans regularly encounter and pass on factually incorrect or misleading claims that are aligned with their political views (3, 6–8). There are two possible reasons why belief expression might correlate with ideological alignment (9, 10): First, people might insincerely express a belief in what they would like to be true even when they know it is false (e.g. “cheerleading”) (11–14). A second explanation is that partisans are honest, either because they differ in what they believe or because they do not know what is true and resolve this doubt in favor of their ideology (“motivated reasoning”) (1, 9, 10, 12, 15, 16). Determining the sincerity of partisan bias is crucial as this differentiates between the misinformation policy challenge being one of merely inducing good behavior vs. one of correcting underlying factual beliefs.

Previous studies have sought to empirically distinguish between insincere and sincere partisan differences by providing monetary incentives for accuracy in participants’ veracity assessment of preexisting news headlines and claims about politics and the economy. Incentives have been found to consistently shrink partisan disagreement, though to an extent that varies substantially across studies (11, 12, 17–22). This evidence suggests that when people express a belief in statements at odds with known facts they are often insincere.

What we problematize here is that past research has primarily worked with preexisting claims that have already circulated in the media, on which official figures are prominently published, or on which opinions by others have previously been expressed. However, many statements encountered online or in casual conversation constitute new claims that are difficult to evaluate or intuit. This is especially true when these statements are encountered before they have gone viral and have undergone fact-checking. It thus remains largely an open question whether people are ideologically biased in their assessment of the veracity of new (mis-)information.

When evaluating the truthfulness of a statement that has already been addressed by experts, the truth will often be obvious. Incentives can then correct insincere support for a falsehood. When in doubt, individuals must decide which sources to believe in. Residual partisan differences in belief may then arise from motivated advice-taking and greater placement of trust in sources that are ideologically aligned (18, 23–25).

Conversely, when content is new, there is a lack of veracity information, so individuals must rely on their personal judgment. When deciding whether a previously unseen claim is likely true or false, individuals will have little else to draw on but their own beliefs in and knowledge of related claims that have received news coverage. In such cases, the extent of residual partisan differences under accuracy incentives will largely depend on individuals’ world views. Substantial partisan differences may persist if individuals place greater trust in sources supporting related claims or use cognitive shortcuts such as party endorsements of related policies (23, 24). Accuracy incentives, designed to encourage correct responses, will have limited impact because they cannot correct ideological differences in beliefs unless individuals themselves are aware of it.

We therefore argue that in the case of previously unseen claims, any partisan differences individuals will exhibit in their veracity assessment will be largely resistant to accuracy incentives. We report on three studies in which participants identifying as either liberal or conservative judged empirical claims of a sensitive political nature that were either in line with or at odds with social science evidence. In contrast to previous studies, these studies systematically confronted participants with newly constructed unfamiliar statements based on evidence they likely did not know of.

## Study 1

We selected 20 empirical results from academic journals most people were unlikely to be informed about. The results pertained to politics, society, and science, and were sufficiently politically charged to be consistent with either a liberal or conservative ideology. We summarized each finding in a text of the length of a tweet. Half of the messages were then modified to contradict the empirical result. Henceforth, we refer to unmodified messages as “true” and modified messages as “false.” The purpose of contradicting select scientific evidence in half the messages was to provide an honest ground for determining accuracy-based payoffs. Equality in the number of true and false statements also neutralizes any acquiescence bias (26) favoring agreement over disagreement.

In an experiment resembling a news recommender system, we exposed 350 US crowd workers recruited from Amazon’s Mechanical Turk platform, known to be strongly driven by a profit motive (27), to the 20 news messages. Participants were asked to “share” a message if they believed it to be true and “discard” otherwise. To assess the impact of accuracy incentives on veracity judgment, we varied the payoff structure across two conditions. Half of the participants received a flat-fee payment (US$1.50). The other half were informed that they would receive a show-up fee (US$1.50) plus a bonus of US$0.10 for each message correctly classified as true or false, and that each message incorrectly classified would reduce their bonus by US$0.10.

**Fig. 1.**
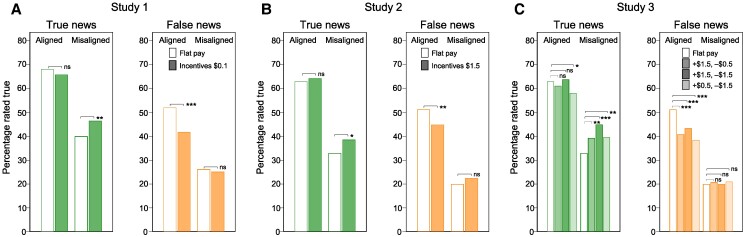
Percentage of messages perceived as true by veracity and ideological alignment. Partisan bias in accuracy assessment remains substantial under monetary incentives: A) Misaligned true messages are assessed as true 19.3±4.7 percentage points less often than aligned true messages, while aligned false messages are judged true 16.6±4.6 percentage points more often than misaligned false messages. This pattern persists if we increase incentives 15-fold (B) and encourage or discourage true ratings when lacking confidence (C). ns: P≥0.05, * P<0.05, ** P<0.01, *** P<0.001.

Our results show that a portion of partisan bias is attributable to insincere beliefs, as it disappeared under incentivization (Fig. 1A). However, the bigger part could not be eliminated and, despite incentivization, partisan bias remained substantial. This suggests that participants misjudged message veracity primarily because their ideology led them to believe it, and they rated true information as false not because they wanted it to be false but because they did not believe it. Incentivization reduced partisan bias—the difference in veracity perceptions between aligned and misaligned news—by 33.5%. Participants correctly assessed aligned true messages significantly more often than misaligned true messages, whether incentivized (aligned: 65.8% vs. misaligned: 46.5%; ATE=19.3, P<0.001, N=1,660) or not (68.1% vs. 40.0%; ATE=28.2, P<0.001, N=1,840). Bias in veracity judgments of true messages—the difference between white and green bars in Fig. 1A, left panel—is reduced by 31.5% (=100%—[65.8%–46.5%] / [68.2%–40.0%]). Vice versa, participants incorrectly assessed aligned false messages significantly more often than misaligned false messages, both when incentivized (41.8% vs. 25.2%; ATE=16.6, P<0.001, N=1,660) and when not (52.1% vs. 26.2%; ATE=25.9, P<0.001, N=1,840). Bias reduction in the assessment of false messages—the difference in difference between white and orange bars in Fig. 1A, right panel—corresponds to 35.7%.

## Study 2

Study 1 leaves open the possibility that participants persisted in their bias because a US$0.10 incentive was too weak. In a second study, we increased incentives 15-fold and informed participants their bonus would increase (decrease) with US$1.5 for each correct (incorrect) decision. With monetary stakes at US$3 per decision, these incentives surpass those in prior studies (11, 12, 19). The control condition’s flat-fee payment was US$3. For study 2, we switched to the Prolific platform, which recent research finds has the highest quality participants (28). Four hundred and two US participants assessed the veracity of the same 20 messages as in study 1.

Again we found incentives to only moderately reduce bias, no more so than in study 1 (Fig. 1B): Incentivization reduced partisan differences in accuracy assessment by 21.6%. As in study 1, only assessments of misaligned true messages and aligned false messages were affected by the treatment. Incentives reduced bias by 14.4% for true news (100%—[64.3%–38.6%] / [63.1%–33.0%]), and by 28.6% for false news (100%—[44.8%–22.5%] / [51.2%–20.0%]). These findings reveal substantial partisan differences in factual beliefs that compromise participants’ ability to take advantage of an unusually good earnings opportunity. The residual bias led participants to forego an average of 57% of their bonus, amounting to a loss of US$7.2 (Supporting Information).

## Study 3

Studies 1 and 2 leave open two theoretical possibilities. The first is that liberal and conservative participants were unsure how to classify many messages and used their ideological priors as tiebreakers. If so, then the partisan differences might largely disappear in a context where expressions of belief in a statement when uncertain are discouraged. Risk preferences might then lead many participants to err on the side of not expressing a belief in a claim when they are not confident. The second theoretical possibility is that participants formed their beliefs with confidence. Study 3 differentiates between these possibilities by varying the reward for correctly rating a true statement as true relative to the punishment for wrongly rating a false statement as true, while keeping the compensation for rating a statement false constant at 0 regardless of its veracity (i.e. individual payoff remained unaffected by any message rated false). Study 3 implements three incentives regimes: encouraging (+US$1.5 for rating true messages true, −US$0.5 for rating false messages true), neutral (+US$1.5, −US$1.5), and discouraging (+US$0.5, −US$1.5). 592 US participants from Prolific partook in study 3.

As in studies 1 and 2, incentives significantly increased the correct classification of true misaligned messages and decreased the incorrect classification of false aligned messages, but left substantial residual bias in perceived accuracy (Fig. 1C). Accuracy incentives reduced partisan bias for true messages by 27, 37, and 39% in respectively the encouraging, neutral, and discouraging condition. For false messages, bias reductions were respectively 36, 25, and 44%. Despite these reductions, participants found misaligned true messages significantly more often false than aligned true messages (P<0.001) in all three incentive conditions. The same is observed for false messages (P<0.001). These results indicate that participants did not use ideology to resolve a difficult choice under high uncertainty, or else they would have used the safe option of not sharing more often in the neutral and discouraging conditions. Rather, the evidence suggests participants formed beliefs consistent with their ideology and many of their veracity assessments were confidently made.

## Discussion

The results show that strong incentives for accuracy fail to correct most ideological differences in the veracity judgment of new (mis-)information. This ideological bias in expressed beliefs is resistant to incentives that discourage the judgment of any statements as true when in doubt. These findings suggest that when confronted with previously unseen claims, individuals form beliefs that are biased in the direction of their ideological leaning. Liberals and conservatives disagree in their expressed belief in newly seen factual statements not because they are pretending, but because their beliefs are formed in alignment with their ideology.

Four limitations circumscribe these results. First, the social science evidence we used to determine participant payment may be uncertain, contradicted in newer studies, or may itself suffer from bias, e.g. toward researcher ideology or positive results. For example, message 15 claiming a decline in undocumented migrants (see Table S1) was true some years ago, but the recent trend has changed. Our main result that partisan bias in expressed beliefs persists under strong accuracy incentives is however robust to changes in what we count as true, because we measure bias as the difference in how an average conservative and an average liberal classify a message. This quantity remains unaltered when we change the classification of messages as true or false. Second, participants may have tried to make decisions concordant with what they expected social scientists to hold true even if discordant with what they themselves hold true. In this light, the ideological differences in factual beliefs revealed here should be treated as a lower bound estimate of the true variation of beliefs among our participants (9). Relatedly, the incentivization effect could be artificial if it led participants to look up the correct answers online. While we cannot completely rule out this possibility (Supporting Information), we did not observe the major reduction in partisan bias one would then expect. Third, the samples of online crowd workers recruited from MTurk and Prolific are not representative of the US population, e.g. conservative participants are less conservative than those in the US population (28). Lastly, the distinction we have drawn between the evaluation of old information studied in prior work and new information studied here is not absolute. Although the statements in our studies were new, participants might have encountered related statements before and held preexisting beliefs. Thus, the limited effects of incentivization may stem from reliance on ideologically familiar sources and partisan advice-taking rather than the assessment of an entirely new claim in line with existing beliefs (6, 23, 25).

These limitations notwithstanding, the results call into question the effectiveness of accountability measures targeted at users of social media platforms (29, 30) when veracity is difficult to gauge. Study 3, which resembled the context of actual platforms by providing a safe “no share” option with minimal consequences for not sharing true content, showed that interventions discouraging the sharing of doubtful information are ineffective. Although we know from the accuracy prompt literature (6) that merely drawing attention to the concept of accuracy can reduce misinformation sharing, accountability interventions can only correct assessments that are made in bad faith. If misinformation is taken for true information due to strong ideological beliefs instead, then incentives for good behavior may only have a modest impact on the truthfulness of the information individuals decide to share.

## Supplementary Material

pgae506_Supplementary_Data

## Data Availability

The data and code that support the findings of this study are available for download at the Open Science Framework: https://osf.io/qwg7e/
