# Supplementary material for: Partisan belief in new misinformation is resistant to accuracy incentives
Source: PNAS Nexus. 2024 Nov 11;3(11):pgae506. doi: 10.1093/pnasnexus/pgae506 (PMC11574615; doi:10.1093/pnasnexus/pgae506)
Supplement: pgae506_Supplementary_Data [file pgae506_supplementary_data.pdf]

Supporting Information Text

Ethics, pre-registration, and replication. The design was approved by the ethics board of the European University Institute, Italy. Pre-registrations as well as all data for replication are available at the Open Science Framework: https://osf.io/qwg7e/.

Recruiting. To recruit participants for study 1, we posted an advertisement for a 3-minute survey on Amazon Mechanical Turk (AMT) and sent a recruitment email to a mailing list of AMT participants in previous experiments. In the survey, US participants were asked to self-identify as either conservative, moderate, or liberal (see below) and leave their platform ID. All individuals who completed the survey received a compensation of $0.45, and all participants who identified as conservative or liberal received an invitation to participate in our experiment. For studies 2 and 3, we used the Prolific platform, which recent research found to have the highest quality participants (1). We used Prolific's pre-screening function to recruit US participants who self-identified as either liberal or conservative. Across studies, advertisements were only visible to US participants with an approval rating of at least 95%, i.e., platform users who had completed at least 95% of previous tasks successfully. We prevented multiple participation and participation across studies by tracking participants' platform IDs. Because we cannot match IDs across platforms, studies 2 and 3 hosted on Prolific were only made available to individuals without AMT accounts.

Data collection. 456 US crowd workers from Amazon Mechanical Turk (study 1) and 994 from Prolific (studies 2 and 3) completed the experiments. Each participant received an overview of tasks, was informed about duration and payment, and was asked for consent. We include screenshots of the general instructions, informed consent, and participant experience during the experiment in Figures S1–S3. Data were collected during May 5–30, 2020 (study 1), Nov. 6–20, 2023 (study 2), and Nov. 23–29, 2023 (study 3).

Participant ideology. In each study, we measured participants' self-reported ideology twice. First, we recorded participant ideology either through a short survey (study 1) or using Prolific's built-in screening filters (study 2 and 3). Upon entering the experiment, we asked participants to indicate their ideology again, using a standard 7-point scale of ideological identification ranging from extremely liberal to extremely conservative (2). Participants who reported a different ideology at the start of the experiment than during pre-screening were excluded from further participation and received a $0.20 compensation.

Data quality. We implemented additional measures to ensure data quality. First, we excluded all data from participants who responded to any message in less than three seconds on three or more occasions. Second, we excluded participants who took more than 30 minutes to complete their task. Studies 2 and 3 further excluded participants who failed at least one of the following three attention check messages: "Europe is in the southern hemisphere," "Mammals need oxygen to breathe," and "Hillary Clinton is the current president of the United States." Before starting data collection, we informed all participants about the exclusion criteria. Of the 1,582 participants who provided informed consent, 132 (8.3%) were excluded: 58 (3.7%) for failing at least one attention check question, 54 (3.4%) for unreasonably short reaction times, 17 (1.1%) for not answering all messages, withdrawing consent, or timing out, and 3 (0.1%) for having a different ideology than previously indicated. With 6.96 seconds per message, the median evaluation time lay well within what we had estimated to be a reasonable time to read and assess messages carefully. Only 3.9% of evaluations took more than 30 seconds. The low percentage suggests that the observed incentivization effect cannot be fully artificial, i.e., that respondents, to obtain the financial reward, simply looked up the correct answers online. We did not record participant demographics to comply with institutional review board and data protection agreements. However, we can further analyze our data by subgrouping based on whether participants identified as 'extremely' conservative or 'extremely' liberal, focusing on their partisan bias in rating ideologically aligned and misaligned messages. Indeed, extreme identifiers more often rated aligned messages as true compared to those with weaker partisan identification, regardless of incentives (incentivized: ATE=9.16, P <0.001, N=9,570; flat-fee payment: ATE=6.40, P <0.001, N=3,870). Similarly, extreme identifiers were less likely to rate misaligned messages as true, both when incentivized (ATE=5.59, P <0.001, N=9,570) and when not (ATE=9.01, P <0.001, N=3,870). These subgrouped results align with theoretical expectations and serve as an additional quality check of our data.

Duration and payment. We restricted the duration of the experiments to 30 minutes. In line with the expectation that incentivation increases participant effort, average evaluation time per message rose from 8.85±0.34 in the flat payment condition to 10.10±0.18 seconds in the incentive conditions (ATE=1.25, P<0.001, N=26,880). Participant pay grossed up to US$11.3 per hour ([60/8] × US$1.5) in the flat payment condition of study 1 and US$22.5 in study 2. In the incentivized conditions, hourly wages would have been US$14.7 (study 1), US$52.6 (study 2), and US$34.4 (study 3), respectively.

Units of analysis. Our sample encompasses 662 self-identified conservatives and 682 self-identified liberals who rated the perceived veracity of 20 messages, leading to 26,880 rating decisions, our units of analysis. Recruiting for study 1 left us with more liberal (281) than conservative participants (175). In accordance with pre-registration, we balanced participant numbers by drawing a random sample of 175 liberals. The modest bias reduction through incentivization found in the sample is comparable to the mean over 100 random samples (–32.1% for true messages; –37.0% for false messages), and larger than in the original unbalanced sample (–25.2% and –23.1%, respectively). The materials required to reproduce our results with the unbalanced sample are available in the replication package at https://tinyurl.com/OSFpackage.

Statistical analysis. Following pre-registration, our central outcome variable is the *percentage of messages rated as true*. This is calculated by taking the number of decisions where participants rated messages as true and dividing it by the total number of rating decisions within a given experimental condition. We analyze this quantity separately for true and false messages, and also for situations where the participant and the message were ideologically aligned versus misaligned. We assess the level of bias exhibited by participants within any experimental condition by calculating the difference in the percentage of aligned messages rated as true compared to the percentage of misaligned messages rated as true:

$$Bias = \frac{Number\ aligned\ messages\ rated\ true}{Number\ aligned\ rating\ decisions} - \frac{Number\ misaligned\ messages\ rated\ true}{Number\ misaligned\ rating\ decisions}$$

To determine whether monetary incentives reduce partisan bias, we compare the differences in percentages between the control condition c (where participants received a flat-fee payment) and an experimental condition t (where participants' responses where incentivized):

$$Bias\ reduction = 1 - \frac{Bias_c}{Bias_t}$$

For statistical inference, we use two-sided random permutation tests with 100,000 repetitions, comparing the percentage of aligned messages rated as true with those of misaligned messages and using participants' decisions as underlying units of analysis. We use permutation tests because they are robust to correlated observations, which is particularly important for our studies because our units of analysis—rating decisions—are nested within participants.

Messages. To create news messages with an evidentiary basis that participants would not readily know, we reviewed articles in general science and social science journals (3). From these, we selected 72 empirical results that were politically charged, aligning with either a liberal or conservative ideology. The topics of these messages included politics, society, and science. We summarized each finding into a tweet-length text. Half of the messages were then altered to contradict the original empirical results by inverting or negating their meanings. This left us with 72 true-false pairs, or 144 messages in total. We calibrated the selection of messages used in the experiment through a pretest involving 497 liberal and 488 conservative AMT workers. Each participant received a random set of 24 messages (out of the 144 total) and was asked to "share" each message based on its perceived veracity (or "discard" otherwise). Each participant only received either the true or false version of any given message. This pretest allowed us to rate messages according to their perceived veracity and ideological bias. We calculated the perceived veracity as the total number of "shares" from both liberal and conservative pretest participants. We defined ideological bias as the absolute difference in perceived veracity between liberals and conservatives. We selected true (false) messages that scored high (low) on perceived veracity and had strong ideological bias. Table S1 lists the 20 messages used. In the last column, we list the academic article that provided the evidentiary basis for the respective message. In studies 1–3, messages appeared in random order on participants' screens. Participants were asked to evaluate each message and "share" it if they believed it to be true, or "discard" otherwise. Either decision removed the message from the participant's inbox. Note that in study 1 the original set of statements consisted of 32 messages. Because of the substantial increase in incentives in studies 2 and 3, we selected a sample of 20 messages to prevent exorbitant data expenses. This sample was then used for comparison across the three studies. In accordance with our pre-registration, the message sample was selected in such a way that study 1 exhibits the same average reduction in partisan bias compared to the full message set being used, thus guarding against stacking the deck in favor of messages where bias cannot be reduced. Our main result of a modest bias reduction through incentivation holds similarly when using the full message set in study 1: –35.4% for true messages and –37.3% for false messages, respectively.

Forgone earnings. In study 1, 61.3% of decisions were correct on average in the incentivized condition. If participants had correctly identified misaligned true messages at the same high rate as aligned true news, and had identified aligned false news at the same low rate as misaligned false news, their bias would have been completely eliminated. In such a case, 70.3% of decisions would have been correct. Given that participants' expected bonus was US\$0.45 = (20 messages × 0.613 × US\$0.10) — (20 × 0.387 × US\$0.10), an increase in accuracy to 70.3% would have resulted in an expected bonus of US\$0.81. In other words, partisan bias led participants to forgo 44.4% of their income. In studies 2 and 3, following the same calculation, participants forwent 57.4% and 54.0% of their bonuses, respectively.

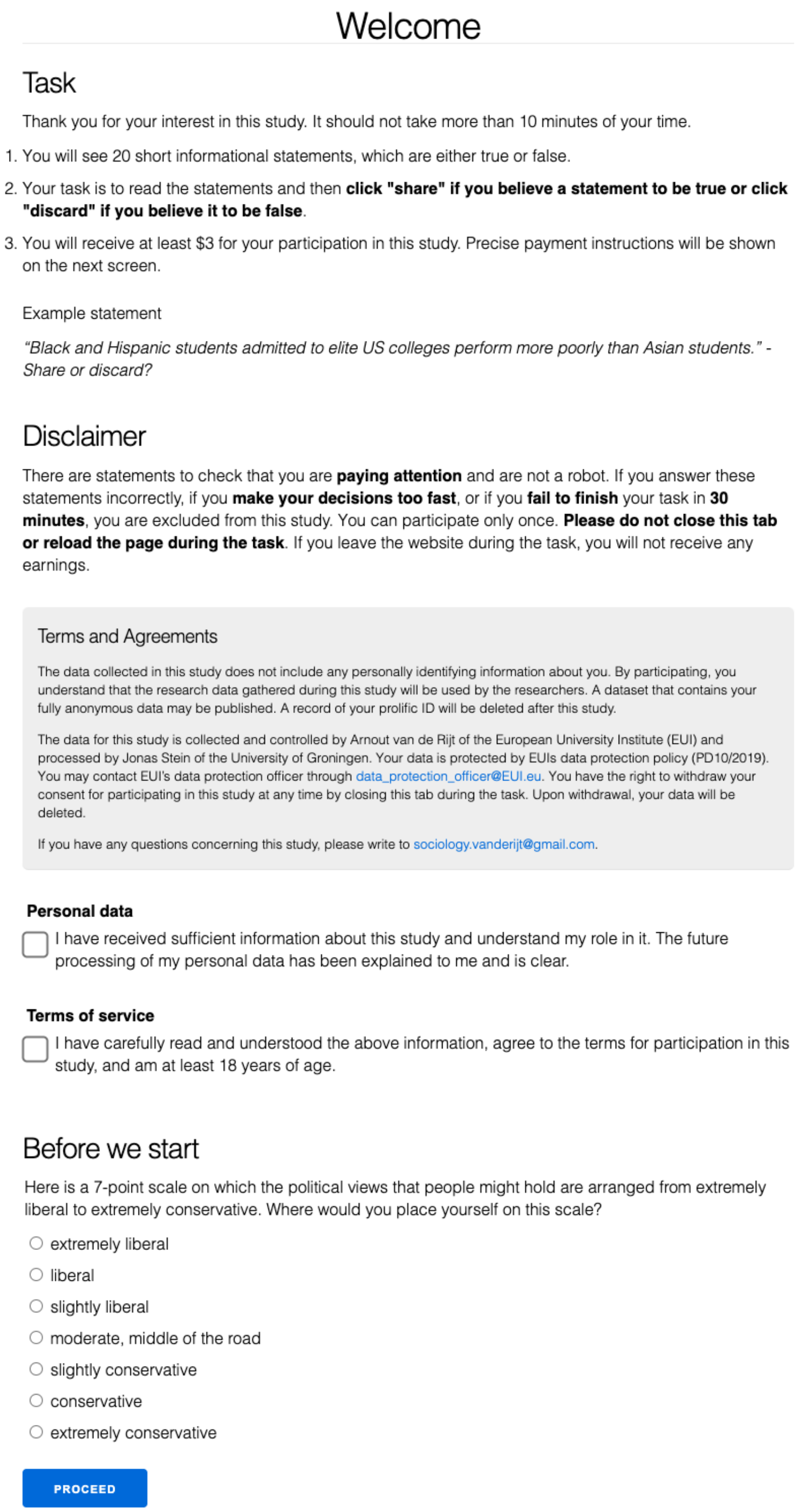

Welcome

Task

Thank you for your interest in this study. It should not take more than 10 minutes of your time.

1. You will see 20 short informational statements, which are either true or false.
2. Your task is to read the statements and then **click "share" if you believe a statement to be true or click "discard" if you believe it to be false**.
3. You will receive at least $3 for your participation in this study. Precise payment instructions will be shown on the next screen.

Example statement

“Black and Hispanic students admitted to elite US colleges perform more poorly than Asian students.” - Share or discard?

Disclaimer

There are statements to check that you are **paying attention** and are not a robot. If you answer these statements incorrectly, if you **make your decisions too fast**, or if you **fail to finish** your task in **30 minutes**, you are excluded from this study. You can participate only once. **Please do not close this tab or reload the page during the task**. If you leave the website during the task, you will not receive any earnings.

Terms and Agreements

The data collected in this study does not include any personally identifying information about you. By participating, you understand that the research data gathered during this study will be used by the researchers. A dataset that contains your fully anonymous data may be published. A record of your prolific ID will be deleted after this study.

The data for this study is collected and controlled by Arnout van de Rijt of the European University Institute (EUI) and processed by Jonas Stein of the University of Groningen. Your data is protected by EUIs data protection policy (PD10/2019). You may contact EUI's data protection officer through data_protection_officer@EUI.eu. You have the right to withdraw your consent for participating in this study at any time by closing this tab during the task. Upon withdrawal, your data will be deleted.

If you have any questions concerning this study, please write to sociology.vanderijt@gmail.com.

Personal data

☐ I have received sufficient information about this study and understand my role in it. The future processing of my personal data has been explained to me and is clear.

Terms of service

☐ I have carefully read and understood the above information, agree to the terms for participation in this study, and am at least 18 years of age.

Before we start

Here is a 7-point scale on which the political views that people might hold are arranged from extremely liberal to extremely conservative. Where would you place yourself on this scale?

○ extremely liberal
○ liberal
○ slightly liberal
○ moderate, middle of the road
○ slightly conservative
○ conservative
○ extremely conservative

PROCEED

Fig. S1. Welcome page showing general instructions, the consent form, and the 7-point scale of ideological self-identification.

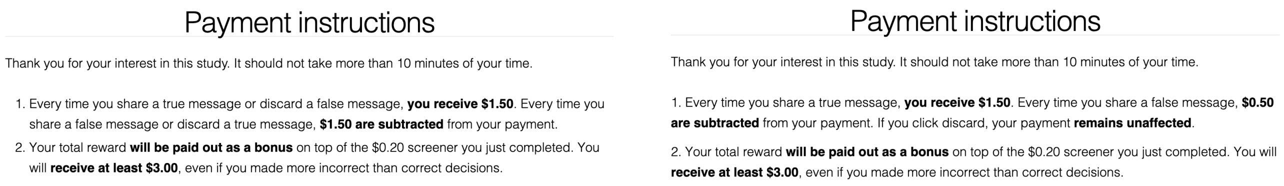

Fig. S2. Payment instructions under high incentives in study 2 (*left*) and in the encouraging condition of study 3 (*right*).

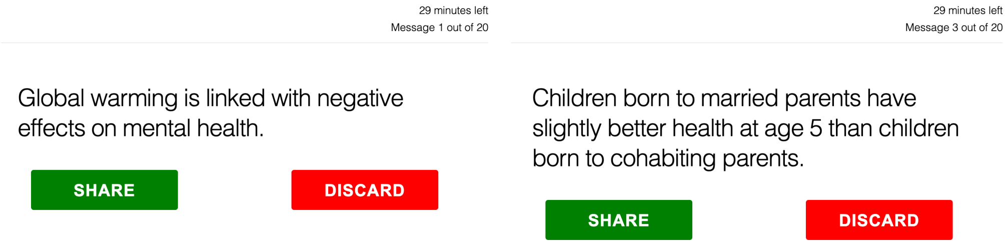

Fig. S3. Messages appeared one after the other on participants' dashboards. The two examples include a true liberal leaning message (*left*) and a true conservative leaning message (*right*).

Table S1. Messages.

	Message	Leaning	Veracity	Source
1	Children born to married parents have slightly better health at age 5 than children born to cohabiting parents	conservative	evidenced	(4)
2	Due to leading professionals and technological advantages, cancer patients in the US have better survival chances than in most European countries	conservative	evidenced	(5)
3	Following Israel's construction of a southern border wall between 2010 and 2013, annual numbers of illegal crossings declined	conservative	evidenced	(6)
4	In countries run by left-wing political parties, immigrants are more likely to be worse at speaking the official language of the new country	conservative	evidenced	(7)
5	Sexual infidelity is more common among unmarried couples who live together than among married couples	conservative	evidenced	(8)
6	Children raised by homosexual parents experience more mental health issues than children raised by heterosexual parents	conservative	contradicted	(9)
7	Foreigners burden the German federal budget, causing a negative balance	conservative	contradicted	(10)
8	Police officers speak with equal respect to all community members, regardless of race, in stop-and-frisk encounters	conservative	contradicted	(11)
9	Scientists have observed that Arctic sea-ice loss has no association with human CO_2 emissions	conservative	contradicted	(12)
10	US states with the death penalty have had much lower homicide rates throughout history as compared to US states without the death penalty	conservative	contradicted	(13)
11	Global warming is linked with negative effects on mental health	liberal	evidenced	(14)
12	Human-induced CO_2 levels in the air have resulted in increased chances of wildfires in California	liberal	evidenced	(15)
13	The greater the immigrant diversity in a city, the higher the wages for white-collar jobs	liberal	evidenced	(16)
14	The greater the share of immigrants in a Western country, the more accepting native citizens are of new immigration	liberal	evidenced	(17)
15	The number of undocumented migrants attempting to cross the US–Mexico border has steadily declined over the past two decades	liberal	evidenced	(18)
16	Affirmative action students from public universities graduate with better grades and in more prestigious fields of study than regular students	liberal	contradicted	(19)
17	Businesses owned by women are much more successful than comparable businesses owned by men	liberal	contradicted	(20)
18	Europe leads the ranking for the best ranked universities in the world	liberal	contradicted	(21)
19	Married people are much more likely to suffer from depression than single people	liberal	contradicted	(22)
20	Men in same-sex relationships are much more likely to be in relationships that are serious, monogamous, and sexually exclusive	liberal	contradicted	(23)

References

1. B. Douglas, P. Ewell, and M. Brauer. Data quality in online human-subjects research: Comparisons between MTurk, Prolific, CloudResearch, Qualtrics, and SONA. *Plos one*, 18(3):e0279720, 2023.
2. American National Election Studies. Cumulative data file codebook, https://electionstudies.org/data-center, 2019.
3. J. Stein, M. Keuschnigg, and A. van de Rijt. Network segregation and the propagation of misinformation. *Sci. Rep.*, 13 (1):917, 2023.
4. K. Schmeer. The child health disadvantage of parental cohabitation. *J. Marriage Fam.*, 73(1):181–193, 2011.
5. M. Coleman et al. Cancer survival in five continents: a worldwide population-based study. *Lancet Oncol.*, 9(8):730–756, 2008.
6. E. Flores. Walls of separation: An analysis of three 'successful' border walls. *Harvard Int. Rev.*, 38(3):10–12, 2017.
7. R. Koopmans. Multiculturalism and immigration: A contested field in cross-national comparison. *Ann. Rev. Sociol.*, 39: 147–169, 2013.
8. J. Treas and D. Giesen. Sexual infidelity among married and cohabiting Americans. *J. Marriage Fam.*, 62(1):48–60, 2000.
9. C. Patterson. Children of lesbian and gay parents. *Child Dev.*, 63(5):1025–1042, 1992.
10. H. Bonin. Der Beitrag von Ausländern und künftiger Zuwanderung zum deutschen Staatshaushalt. *Bertelsmann Stiftung*, pages 1–71, 2014. .
11. R. Voigt et al. Language from police body camera footage shows racial disparities in officer respect. *Proc. Natl. Acad. Sci. U.S.A.*, 114(25):6521–6526, 2017.
12. D. Notz and J. Stroeve. Observed arctic sea-ice loss directly follows anthropogenic CO2 emission. *Science*, 354(6313): 747–750, 2016.
13. C. Manski and J. Pepper. Deterrence and the death penalty: Partial identification analysis using repeated cross sections. *J. Quant. Criminol.*, 29:123–141, 2013.
14. N. Obradovich, R. Migliorini, M. Paulus, and I. Rahwan. Empirical evidence of mental health risks posed by climate change. *Proc. Natl. Acad. Sci. U.S.A.*, 115(43):10953–10958, 2018.
15. E. Wahl, E. Zorita, V. Trouet, and A. Taylor. Jet stream dynamics, hydroclimate, and fire in California from 1600 CE to present. *Proc. Natl. Acad. Sci. U.S.A.*, 116(12):5393–5398, 2019.
16. A. Cooke and T. Kemeny. Cities, immigrant diversity, and complex problem solving. *Res. Policy*, 46(6):1175–1185, 2017.
17. E. Schlueter, A. Masso, and E. Davidov. What factors explain anti-Muslim prejudice? An assessment of the effects of Muslim population size, institutional characteristics and immigration-related media claims. *J. Ethn. Migr. Stud.*, 46(3): 649–664, 2020.
18. D. Massey, J. Durand, and K. Pren. Explaining undocumented migration to the U.S. *Int. Migr. Rev.*, 48(4):1028–1061, 2014.
19. A. Dias Lopez. Affirmative action in Brazil: How students' field of study choice reproduces social inequalities. *Stud. High. Educ.*, 42(12):2343–2359, 2017.
20. A. Robb. Entrepreneurial performance by women and minorities: The case of new firms. *J. Dev. Entrep.*, 7(4):383–397, 2002.
21. N. Bowman and M. Bastedo. Anchoring effects in world university rankings: exploring biases in reputation scores. *High. Educ.*, 61:431–444, 2010.
22. P. Amato. Marriage, cohabitation and mental health. *Fam. Matters*, 96:5–13, 2014.
23. K. Joyner, W. Manning, and B. Prince. The qualities of same-sex and different-sex couples in young adulthood. *J. Marriage Fam.*, 81(2):487–505, 2019.
